# The Laurentian University Library and Archives and the Covid-19 pandemic

**DOI:** 10.1177/0955749020982898

**Published:** 2020-08

**Authors:** Brent C Roe

**Affiliations:** 7728 Laurentian University, Sudbury, Ontario, Canada

**Keywords:** archival services, Covid-19, Laurentian University, library management, library services, pandemic

## Abstract

The University Librarian at Laurentian University in Sudbury, Ontario, Canada, describes the experience and actions of the Laurentian University Library and Archives in the context of the Covid-19 pandemic over the spring and summer of 2020. The sudden shutdown of the university campus in March 2020 entailed financial strains that had direct consequences for library management, affecting operations, collection access and staffing. The shutdown itself was an extreme disruption to the provision of library and archival services. The pandemic and the campus shutdown were experienced as disorienting and stressful for library employees, although work continued as well as possible. Over the course of the summer, a kerbside pickup and scanning service was launched, a HathiTrust membership was sought, on-site archival work was restarted and plans were prepared for opening library space for study.

Laurentian University (www.laurentian.ca) is a medium-sized (some 6,800 full-time-equivalent students), bilingual (English and French), public university in Sudbury, Ontario, Canada, at about a 5-h drive north of Toronto. As the University Librarian at Laurentian, it is, in fact, somewhat at second hand that I recount how the Laurentian University Library and Archives (www.laurentian.ca/library) dealt with the Covid-19 crisis, at least in the early spring of 2020: I had begun what was to be a 6-month administrative leave on 1 March and was far from the events both physically (I was in Japan) and, to a lesser extent, mentally. I was, nevertheless, reading my email.

On the morning of Wednesday, 11 March, we learned that Sudbury had its first case of Covid-19: a man who had returned from a conference in Toronto became ill – alas, he worked in a shared government-university building on the edge of campus. At first, just this building was closed, but other campus operations and classes continued as normal. In the library, there were questions from librarians and support staff members as to library plans if there were a need to close the campus. At this point, I need to introduce my colleagues Lace Marie Brogden, Dean of the Faculty of Education and Acting University Librarian at the time, and Ginette Gervais, Operations Manager for the Library and Archives; Dr. Brogden had overall responsibility for the plans of the library and communicated with our seven librarians (including one archivist), and Ms. Gervais was (and is) supervisor of our dozen full-time support staff members and our half-dozen part-time student employees and she communicated with them: so far the message was ‘business as usual’.

By noon the same day, the university declared that all classes were immediately suspended, and courses would henceforth continue online only. While laboratories were closed and campus events that week were cancelled, the university remained open and employees were still to be at work. By the end of the workday, the university clarified for managers that if employees could usefully work remotely, managers could permit staff to work at home; otherwise, staff should continue to work as normal, though taking greater hygienic measures. Most of the librarians, who have more autonomy than the support staff, had already decided that they should carry on with their work at home and online, and Dr. Brogden affirmed that they could indeed do so in an evening message.

While Laurentian University was the first university in Canada to have suspended its on-campus classes because of Covid-19 concerns, there were already circulating on listservs among Canadian libraries various Covid-19 resources and spreadsheets of measures being taken at university libraries. In the days following 11 March, other Canadian universities suspended on-campus classes and began modifying their library services. At Laurentian, a number of decisions were quickly made: borrowers should keep their books until further notice, with a suspension of overdue fines; the interlibrary loan service would request and provide only born-digital items; all reference assistance would be conducted virtually; all library instruction would occur virtually; and all archival service would be suspended (at least that which required consultation of archival documents). As Canadian universities were now forbidding non-essential work-related travel even within Canada, various library-related meetings were now being cancelled or moved online; for example, the National Forum of the Canadian Federation of Library Associations (CFLA), which was to occur in April in Winnipeg, Manitoba, and for which I was Co-Chair of the planning committee, was cancelled on 13 March by the CFLA Board.

As of Friday, 13 March, the library weekday closing time was changed from 11:00 p.m. to 4:30 p.m. While the librarians were already working remotely, most of the support staff members were continuing to work on-site. There was email discussion among members of the university’s Joint Occupational Health and Safety Committee as to whether the library should close entirely or remain open with its reduced hours and greatly reduced traffic (on 12 March, there were ‘less than 30’ patrons entering the library). Independently of that discussion, the Acting University Librarian informed library employees that the library would remain physically closed from the end the day until further noticed. Ironically, the day before, the building linked with the first Covid-19 case at the university and in the city had been reopened for regular activity after being ‘thoroughly disinfected’.

According to the library Operations Manager, most of the support staff members were expressing a preference for continuing to work on-site at that point since their regular work would normally require this for practical reasons. Nevertheless, aside from herself and a couple of others needed to attend to library shutdown details, they were now expected to work from home. These staff members included two circulation clerks, four library assistants, four library technicians, one archival assistant and the administrative assistant. The Operations Manager began a series of calls by Zoom that she called team ‘chats’ (not ‘meetings’) that were an effort not only to deal with practical work-related matters but also were a means of preserving a sense of connection with their place of work and with each other as colleagues. She encouraged individuals to share their concerns and coping strategies in these new and uncertain circumstances, but she also encouraged more positive and normalising conversation on, for example, family pets and spring gardening plans. Staff members were also asked to submit a daily summary of their work.

While it was true that the amount of practical work that several of the support staff members could accomplish was limited because very little could be done at a distance (say, by our circulation clerks), she encouraged staff members to take the opportunity to undertake learning activities that might be generally useful for their work. For example, one staff member spent time learning, at last, how to use Google Drive, Docs and Sheets; another took the opportunity to learn more fully library terminology in French; yet another attended a whole 3-day conference online that dealt with statistical and geographic information services.

Aside from staff members doing whatever work they could manage to do from home, and aside from their team chats and learning projects, certain of them had personal concerns that took them understandably somewhat ‘off-task’: small children, who were soon sent home as schools closed, or ageing parents who needed greater assistance as these began to avoid public places, being at especially high risk of Covid-19 complications. Others needed to deal with their own anxiety around their unusual work circumstances, their own health and the security of their employment.

By 20 March, the librarians, working together by Zoom, email and Google Docs, had assembled the text for a full and detailed library FAQ (a ‘frequently asked questions’ response list) to be added to the quickly growing collection of FAQs relating to many other university services and operations. The FAQ alerted library users of the service decisions mentioned above, among other details, and stressed the availability of the librarians to assist with research questions of the students and faculty as well as possible.

Meanwhile, the university, already experiencing a challenge in arriving at a balanced budget to present to its Board of Governors, began to tabulate the various new costs (e.g. enhanced cleaning), lost revenues (e.g. from ancillary services) and potentially lower enrolment in summer and fall classes because of Covid-19. We had already known that the library collections and operations budget would be reduced by some $187,000 (CAD), that is, by almost 10%, but there was a proposal (from ‘above’) that its budget be reduced by up to more than twice this amount to help with the shock to the university’s finances. The Acting University Librarian argued, however, with data provided by the librarians, that this would be unfeasible, given that so large a part of the library collections budget was committed to multi-year licenses or to essential resources that could be dropped only with great harm to campus academic programmes and research. Later in the spring, the university adopted a ‘preliminary budget’ that included only the originally planned (pre-Covid-19) budget cut.

In the wake of the Covid-19 closure, however, the staffing complement of the library was reduced. One full-time employee, who was working on a contract that was due to lapse (though expected to be renewed) later in the spring was laid off temporarily, with certain severance options, as were most contract employees across the campus as a cost-control measure. While it was true that there was no real work for this evening and weekend circulation clerk to perform while the library remained closed, her potentially permanent loss to us would mean that we had no full-time staff member to work in the evening or on weekends once the library eventually reopened (sometime in the fall?), and we had not been in the habit of staffing our evenings and weekend hours with only part-time student help. In any case, all of our six part-time student employees were also laid off, though with some (reduced) pay continuing for them to the end of the winter term (their normal employment end date).

As an additional cost-control measure, the university offered certain early retirement incentives to support staff across the campus. In the end, this meant, for the library, that we lost to retirement one of our three general library technicians (who effect book purchases, book cataloguing, periodicals management and various collection maintenance tasks) and our one inter-library loan (ILL) and document delivery library technician. While we believed that we could manage, going forward, without a dedicated ILL technician, as our general library technicians would be able to incorporate that work (both ILL activity and, of course, our title-by-title book purchasing had been diminishing in recent years), it would be difficult to cope with just two library technicians, although we had only faint hope that we would be given permission to recruit a third library technician any time soon.

One other institutional cost-cutting measure that affected library personnel and operations was, first, a decision by the university administration that all university administrators and managers would have to take a 5-day furlough (unpaid vacation) and, from 1 July 2020, accept a 3% gross salary reduction. In the library, this included me as University Librarian and our Operations Manager. A few weeks later, upon the ratification of a new collective agreement between the university and the union representing the support staff, our support staff members were also asked to take a furlough (6 days for them) and a 1% salary reduction.

By the end of May, as the Covid-19 crisis continued, given that Dr. Brogden, my decanal colleague and Acting University Librarian, had plenty of work with her own faculty, I decided to cut short my administrative leave and return to work as the University Librarian – from my home office – as there was much work to do. Aside from several Covid-19-related projects, which I summarise below, our library had begun in May the time-consuming process of a planned migration from a version of the open-source Evergreen library management system to Ex Libris’ Alma and Primo VE systems in the context of the Omni consortium of Ontario university libraries. In the context of the financial pressures of the university, it was necessary to prepare our fall communication to the campus about that such that it was clear that much of the cost of the migration had been covered in the previous fiscal year and that the new system would facilitate a fuller exploitation of our (soon to be somewhat reduced) information resources.

As one way to improve access to scholarly content in the context of our inability to provide access to our physical collection (at the time, we did not even have any staff physically entering the library), we decided that we would pursue a membership in HathiTrust, under which we would be able to avail ourselves of the HathiTrust Emergency Temporary Access Service (ETAS). This service would allow limited stop-gap online access to many in-copyright titles that Laurentian faculty and students would normally have been able to physically consult or borrow locally. We had assumed that establishing our membership would be a quick process, but it took a couple of months to achieve member access to the HathiTrust collections. The major problem was the need to set up SAML-2.0-based authentication, which we had not used previously at Laurentian, and to make this work with the HathiTrust system.

Meanwhile, while the campus had been very patient over the spring with not having access to our physical collections, there were more and more requests for the use of physical books. Like other libraries at this point, we planned a ‘kerbside pickup’ service whereby, once we had some staff approved to work in the library, patrons could request books that would be charged out to their accounts and delivered to them at the door of the library building. A formal safety plan was needed, incorporating all necessary hygienic measures, and the campus Operational Resumption Committee had to approve it. By the beginning of August, we were pleased to finally be able to launch this service.

Along with the kerbside pickup service, using the same request form, library staff also provided a service whereby individual book chapters or articles from paper journal volumes would be scanned and sent to requesters by email. The scanning had to honour any applicable copyright restrictions, of course, and it remained a challenge for us to provide OCR-compatible PDF files rather than just JPEG files with the equipment we had. Both of these services have proved to be popular. The kerbside pickup service has not been without complexities, especially in relation to incorporating, at least partially, the libraries of our on-campus federated universities and our downtown Architecture Library. We also recognised that we would need to adjust our lending and scanning policies once our access to the HathiTrust ETAS was activated so as to honour HathiTrust copyright-related restrictions.

At the same time as we launched these services, we unlocked our external book return bin, and so started to receive returned books. Once placed on book chariots by our masked and gloved staff, the returned books were to be left in quarantine for 4 days before they would be further manipulated for re-shelving. So as to encourage the return of other borrowed books, we contacted by email all persons with books that they had earlier signed out to offer them a shipping label for the postage-paid return of the books by mail. We applied our usual distance services limits such that persons residing within 50 km of campus were expected to bring their books back themselves (although we were open to making allowances for special cases). It is nevertheless very likely that we will have lost a number books to non-returning borrowers.

The next focus in our operational resumption process was the Archives. Another detailed safety plan and committee approval was required for this, but by early August, the archivist and archival assistant were able to return to on-campus work to address a backlog of archival reference service requests. Limited on-site consultation of archival fonds was anticipated in our plan, principally by academic researchers, and this was supported by the Vice-President Research inasmuch as she viewed the Archives as a laboratory for humanists – and science laboratories across campus were now also re-opening.

As of late August, we are currently developing two additional safety plans. The first plan is to allow more of our support staff to work regularly on campus in order to catalogue new books and perform various collection and shelf maintenance tasks. Our library technicians will also eventually process interlibrary loan and document delivery requests, but at this point we are waiting for a critical mass of other Ontario university libraries to announce themselves ready to recommence these services, at which time the Ontario Council of University Libraries (OCUL) will recommence its logistical support for this shared service.

The second plan is to open, at some point in the fall, the main floor of the main library as a study space, if this should prove needed. At time of writing, it is anticipated that some 500 students will be living in on-campus residences, with the result that some study spaces will be needed even though almost no courses will be offered in physical classrooms, at least during the fall term. Because the main library is relatively far from the residences, and there is a newly renovated non-staffed study lounge much closer, the library will be opened as a study space only once the other study lounge seems to be used at capacity (i.e. with a maximum of 50 students spaced at least 2 m apart). The library, if or when used for study, will also provide only 50 physically distanced study spaces at first, with the possibility at yet a later date of opening an additional floor with another 50 seats. The bookstacks will be cordoned off, although our kerbside pickup service will change to an internal ‘counter-side pickup’ service – ideally before the arrival of the inclement late autumn weather. It should be noted that librarians will continue mostly to work from home, providing library instruction and research consultation by Zoom or other virtual means.

There remains much uncertainty about the near future of the university in the Covid-19 context, both on the enrolment and budgetary front and on the Covid-19 management front. While regionally, the number of Covid-19 cases remains very low, it is possible that new cases will appear among students in the residences or that there will be a ‘second wave’ in the Sudbury area during the coming months. If this occurs, the campus will likely reverse its current movement towards re-opening and normalisation of operations (with, of course, the omnipresent face masks and containers of hand sanitiser). We are taking things week by week and hoping for the best for the 2020–2021 academic year.

